# Idiopathic bronchial hemorrhage: a rare but catastrophic complication in cardiac surgery

**DOI:** 10.1186/s13019-016-0477-0

**Published:** 2016-05-05

**Authors:** Takeshi Uzuka, Masanori Nakamura, Tomohiro Nakajima, Shinichi Kusudoh, Hiroaki Usubuchi, Akihiko Tanaka, Noriyasu Watanabe

**Affiliations:** Cardiothoracic Surgery, Sapporo City General Hospital, Sapporo, Hokkaido 060-8604 Japan; Cardiovascular Surgery, Sapporo City General Hospital, North 11 West 13 Chuo-ku, Sapporo, Hokkaido 060-8604 Japan

**Keywords:** Idiopathic bronchial hemorrhage, Complication, Cardiac surgery, Hemoptysis

## Abstract

**Background:**

Hemoptysis is a common complication in all kinds of surgery. However, it is rarely critical because it resolves with or without intervention.

**Case presentation:**

Here the authors present what is believed to be an unprecedented report of a case involving a fatal idiopathic bronchial hemorrhage complication during cardiac surgery. Eighty-five-year-old female with severe aorticvalve stenosis had elective aortic valve replacement. Subsequently, she developed diffuse bilateral severe idiopathic bronchial hemorrhage which required maximum intervention such as external bronchial ligation, V-A ECMO, coil embolization of bronchial artery and internal airway blockage by spigot.

**Conclusions:**

Airway bleeding is not a rare complication in cardiac surgery, but this case should increase awareness of this potentially life threatening perioperative complication.

**Electronic supplementary material:**

The online version of this article (doi:10.1186/s13019-016-0477-0) contains supplementary material, which is available to authorized users.

## Background

Airway bleeding during or after heart surgery is common, but is rarely reported because it is generally minor and resolves spontaneously in most cases. Here the authors report an unusual case of exsanguinating diffuse airway bleeding during cardiac surgery. The complication required aggressive intervention measures such as extra-corporeal membrane oxygenation (ECMO), bronchial ligation, bronchial artery coil embolization and internal airway blockage using an intrabronchial spigot.

## Case presentation

The case involved an 85-year-old female with congestive heart failure caused by severe aortic valve stenosis. Aortic valve replacement with a bioprosthetic valve was conducted electively. After the ascending aorta was unclamped, anesthetist noticed blood coming up from the endotracheal tube. Intraoperative flexible bronchoscopy confirmed massive hemoptysis coming up from all peripheral bronchi and mainly from the bilateral inferior lobes. The left chest was inspected through additional anterolateral thoracotomy connected perpendicularly to median sternotomy wound at 4th intercostal space. And the left hilum was exposed and lower bronchus was ligated externally to reduce the amount of intrabronchial bleeding. Repeated bronchoscopy showed persisting large-volume airway bleeding (Additional file 1: Video S1) and weaning cardiopulmonary bypass was impossible due to a reduced tidal volume of up to around 50 ml caused by severe peripheral airway obstruction. Groin cannulated veno-arterial ECMO with heparin coated circuit was initiated as a substitute for conventional cardiopulmonary bypass, and heparin was reversed precipitously with full dose of protamine sulfate. To treat the persistent airway bleeding, the chest wound was closed and the patient was shifted to a catheterization laboratory for bronchial artery embolization. Intraoperative total blood loss from the airway was over 4000 ml.

**Fig. 1:**
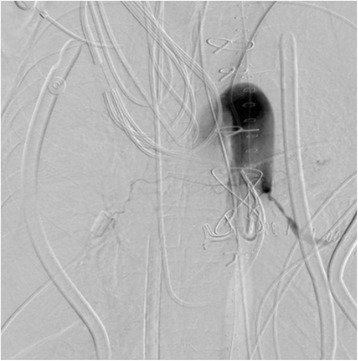
Bronchial artery angiography and embolization. The bronchus in the left lower lobe was enhanced by bronchial artery angiography

Angiography confirmed massive bronchial artery bleeding into the left lower bronchus (Fig. 1). However, diffuse bronchial artery bleeding was observed in the left upper lobe and right lower lobe as well. Embolization of the bilateral bronchial artery was conducted successfully. However, hemoptysis continued and repeated bronchoscopy confirmed ongoing diffuse bleeding. The right lower bronchus was blocked internally with an Endobronchial Watanabe Spigot (Nobatech, Cedex, France). Even after these aggressive interventions, diffuse hemorrhage persisted.

Eventually the amount of hemoptysis reduced on day four, and external ligation of the left inferior bronchus was finally released. The patient’s hemodynamics showed slight improvement on day five with, and a decision was made to convert to Venous-Venous ECMO. However, the patient crashed again and passed away from multi-organ failure on day six.

## Discussion

The term used for the event is idiopathic bronchial hemorrhage. This may overlap but differs from the more documented diffuse alveolar hemorrhage, which is characterized by diffuse microvascular bleeding. In this case, the bronchial artery was responsible for the exanguinating hemoptysis, and no pre-existing lung disease was suggested. Accordingly, the complication was diagnosed as idiopathic bronchial hemorrhage. To the authors’ knowledge, this is the first report of idiopathic bronchial hemorrhage during cardiac surgery, which was diagnosed clearly by bronchoscopy and bronchial angiography.

Most cases of reported exsanguinating hemoptysis (defined as blood loss exceeding 1000 ml at a rate of at least 150 ml/h [1, 2]) have been related to pulmonary artery catheter (PAC) induced trauma [3–6]. Although localization of the site of perforation from PAC is impossible as there is not one connecting tunnel but more or less a series of small bleeding channels, the authors do not believe that PAC injury is the cause of this massive hemoptysis because intraoperative bronchoscopy showed diffuse bleeding from both lungs. For this bronchoscopy finding, we could not isolate or remove the damaged lung.

There is no doubt that systemic heparinization in cardiac surgery triggered the diffuse bronchial hemorrhage. Complete heparin reversal and expeditious weaning from cardiopulmonary bypass is reported to be the best and the only way to manage major hemoptysis [1–5]. However, in this case, due to the massive airway bleeding, circulation and oxygenation could not be maintained without mechanical support. The authors believed that the only following option was to reverse heparin fully and implement bronchial arterial embolization in the catheterization laboratory. However, it would have been effective to block pulmonary artery branch by inflating the balloon of the PAC because there is a certain level of microvascular connection between bronchial and pulmonary circulation [7].

Underlying pulmonary disease such as bronchiectasia or tuberculosis can also cause perioperative hemoptysis. Pulmonary capillaritis such as Wegener’s granulomatosis or Goodpasture syndrome are also known to be related to pulmonary hemorrhage [8]. Although preoperative chest CT and spirometry did not suggest any underlying pulmonary disease, it cannot be excluded because postmortem autopsy was declined by the patient’s family.

## Conclusion

In summary, this was a rare case of diffuse exsanguinating hemoptysis during cardiac surgery. Even after all the aggressive intervention and intensive care administered, the patient’s respiratory status failed to show improvement in terms of lung compliance and oxygenation. Practicing cardiothoracic surgeons should note this unusual but catastrophic perioperative complication.

## Ethics and patient consent

All authors have approved the manuscript. Patient’s family has agreed with submission of the case report. Upon acceptance, authors will transfer copyright to the Publisher. All authors have no ethical problem or conflicts of interest to declare.

## Additional file

Additional file 1: Video S1. Flexible bronchoscopy during the operation. Exanguinating airway bleeding was observed. (MP4 40768 kb)
